# Treatment in the pediatric emergency department is evidence based: a retrospective analysis

**DOI:** 10.1186/1471-2431-6-26

**Published:** 2006-10-06

**Authors:** Kellie L Waters, Natasha Wiebe, Kristie Cramer, Lisa Hartling, Terry P Klassen

**Affiliations:** 1 Department of Pediatrics, University of Alberta, Edmonton, Alberta, Canada; 2 Alberta Research Centre for Child Health Evidence, Department of Pediatrics, University of Alberta, Edmonton, Alberta, Canada

## Abstract

**Background:**

Our goal was to quantify the evidence that is available to the physicians of a pediatric emergency department (PED) in making treatment decisions. Further, we wished to ascertain what percentage of evidence for treatment provided in the PED comes from pediatric studies.

**Methods:**

We conducted a retrospective chart review of randomly selected patients seen in the PED between January 1 and December 31, 2002. The principal investigator identified a primary diagnosis and primary intervention for each chart. A thorough literature search was then undertaken with respect to the primary intervention. If a randomized control trial (RCT) or a systematic review was found, the intervention was classified as level I evidence. If no RCT was found, the intervention was assessed by an expert committee who determined its appropriateness based on face validity (RCTs were unanimously judged to be both unnecessary and, if a placebo would have been involved, unethical). These interventions were classified as level II evidence. Interventions that did not fall into either above category were classified as level III evidence.

**Results:**

Two hundred and sixty-two patient charts were reviewed. Of these, 35.9% did not receive a primary intervention. Of the 168 interventions assessed, 80.4% were evidence-based (level I), 7.1% had face validity (level II) and 12.5% had no supporting evidence (level III). Of the evidence-based interventions, 83.7% were supported by studies with mostly pediatric patients.

**Conclusion:**

Our study demonstrates that a substantial proportion of PED treatment decisions are evidence-based, with most based on studies in pediatric patients. Also, a large number of patients seen in the PED receive no intervention.

## Background

The term "evidence-based medicine" has become a catchphrase for the twenty-first century. Physicians are being called upon to justify their treatment decisions with valid, up-to-date evidence. There have been attempts within many areas of medicine to quantify the evidence that is available to, and used by, the physicians of that discipline. The first such study, conducted by Ellis et al [1] in 1995, found that 82% of treatments in internal medicine were evidence-based. This study has been replicated among a variety of disciplines, including family medicine [2] (81% evidence-based interventions), hematology [3] (70% evidence-based interventions), and surgery [4] (45% interventions based on randomized control trial [RCT] evidence or better).

Within pediatrics, there have been three such studies. Moyer et al [5] examined pediatric inpatients and found that 75% of primary interventions were supported by evidence. Rudolf et al [6] found 40% of clinical actions in a community-based pediatric practice were supported by RCT evidence and 7% were supported by convincing non-experimental evidence. In a study of pediatric surgical patients, Baraldini et al [7] found that only 29% of their treatments were based on level I or II evidence, as defined by Ellis et al (described below).

There is a need to investigate whether and to what extent the treatment of pediatric emergency patients is based on evidence. The wide variety of ailments seen within this area should offer a more comprehensive assessment of the overall state of evidence within pediatrics – encompassing surgical, outpatient and inpatient medicine, across all ages. At the same time, we need to gain an understanding of the quality and quantity of evidence in existence to support pediatric clinical decisions. How often is adult evidence generalized to the treatment of children? This is an important issue given that children differ from adults in terms of their physiology, biology, and developmental processes. There is limited health information available from research in children and, as a result, health care providers repeatedly turn to other sources of evidence, such as adult studies, to assist with managing health care issues in children [8].

In the present study, we sought to identify the extent to which evidence exists for use within the unique environment of the pediatric emergency department. A secondary objective was to determine the amount of evidence, applied to pediatrics, that is derived from adult studies. We hypothesized, a priori, that this was a common practice within pediatrics. Our hope was to identify areas within the body of evidence for pediatric emergency medicine that require future study.

## Methods

This study was approved by the Health Research Ethics Board at our institution.

We conducted a retrospective chart review of randomly selected patient charts based on patients seen in a Pediatric Emergency Department at a tertiary care facility during the 2002 year (January 1 to December 31). The sampling frame was created using the Emergency Department Information System (EDIS) database. The following variables: hospital record number, patient hospital number, patient visit number, date, time, and age made up the sampling frame. A random sample of patient visits were then generated using random-number computer software (S-plus 6.0). Any individual patient was included only once in the sample. A sample size calculation of 295 showed that a minimum of 5.6% precision would be achieved for any given estimate of percentage. The primary investigator (PI) retrospectively reviewed the sampled records and, using a standardized abstraction form, identified a primary diagnosis and primary intervention for each based on the definition set out by Ellis et al. That is, the primary diagnosis was defined as "the disease, syndrome or condition entirely or, if there were several diagnoses, [the diagnosis] most responsible for the patient's [visit to the pediatric emergency department]" [1]. The PI used information from the emergency physician's notes, lab and radiographic results, and any other relevant documentation from the emergency room visit to establish this. If conflicting information was present on the chart, the final diagnosis, as described at the bottom of the emergency record form, was used.

The primary intervention was taken to be "the treatment or other maneuver that represented our most important attempt to cure, alleviate, or care for the patient [with respect to] his or her primary diagnosis" [1]. This was also determined by the PI based on the patient's chart.

A thorough literature search was then undertaken with respect to the primary interventions. Current (at the time of searching) MEDLINE, EMBASE and the Cochrane Library databases were searched comprehensively to determine if any evidence existed with respect to the individual primary interventions. Abstracts and references of relevant scientific papers were also searched. Local experts were contacted and asked if they were aware of RCTs in existence that had not yet been published or that were not found through the search strategy (this step yielded no additional information).

The primary interventions were then divided into the following categories that have been used in previous research [1-4].

### Level I evidence

The abstract or paper demonstrated that this intervention had been evaluated in an RCT, systematic review or meta-analysis. No attempt was made to rate the quality of the study or its findings as this did not relate directly to our goal of determining the quantity of evidence available to PED physicians.

### Level II evidence

No abstract or paper could be found that investigated this intervention in an RCT, systematic review or meta-analysis. However, a committee determined there to be convincing non-RCT evidence for this intervention. That is, the face validity of such an intervention was considered to be so great that randomized trials were unanimously judged to be unnecessary or, if a placebo were to be involved, potentially harmful (i.e., giving a blood transfusion to a patient who was exsanguinating [1]). The committee for this study was made up of five pediatric emergency physicians with extensive experience in pediatric emergency medicine and evidence-based medicine, one of whom was a co-investigator. The remainder of the committee had no other involvement in the study and was provided with only the definitions of the levels of evidence and a brief synopsis of the patient visit. Only unanimous committee decisions were considered sufficient for the inclusion of the intervention as level II evidence.

### Level III evidence

No abstract or paper could be found that involved this intervention in an RCT, systematic review or meta-analysis and the committee did not unanimously agree that there was convincing non-RCT evidence to support it.

Level I evidence was further subdivided into those studies that included a majority (i.e., more than 50%) of pediatric patients and those that did not. Systematic reviews and meta-analyses were included under the "pediatric studies" grouping if they included one RCT of predominantly pediatric patients. Every attempt was made to search out pediatric studies, even if an adult RCT was found first for any given intervention.

A further category was included of those patients who received no primary intervention. This category included patients who were discharged from the pediatric emergency department after receiving only reassurance and/or advice. The percentage of primary interventions which were included in each category was calculated as was the percentage of primary interventions deemed to be from pediatric and adult studies, along with their exact 95% confidence intervals [9].

## Results

From the 18,855 patient visits to the PED during the year January 1, 2002 and December 31, 2002, a random sample of 295 patient charts was computer generated, representing approximately 1% of patient visits. Any individual patient was only included once in the sample. Of these 295 charts, 272 (92.2%) were available to be viewed. The other 23 were missing from the chart area. Of the 272, 10 were discarded because of insufficient information to determine a primary diagnosis and intervention (i.e. the emergency record was missing from the chart). Ninety-four patients received no intervention, representing 35.9% of our sample (95% CI 29.9 to 41.9).

Table 1 lists the interventions that qualified as level I evidence, based on the finding of at least one RCT or SR. In total, 135 patients were found to have received evidence-based interventions (level I evidence), representing 80.4% (CI 73.4 to 85.9) of patients who received interventions and 51.5% of our sample.

**Table 1:** Diagnoses and Interventions seen in the PED for which there is Level I Evidence

**Diagnosis**	**Intervention**	**Number of Patients**	**Reference**
**Evidence from Pediatric Studies**			
msk pain/soft tissue injury	analgesic	17	Clark 2003^[10]^
Asthma exacerbation	inhaled β-agonist	12	Travers 2001^[11]^
radial fracture	reduction & cast	10	McLauchlan 2002^[12]^
otitis media	PO antibiotics	10	Kozyrskyj 2000^[13]^
dehydration (failed PO rehydration)	IV rehydration	6	Atherly-John 2002^[14]^
bronchiolitis	salbutamol	6	Kellner 2000^[15]^
dehydration	PO rehydration	5	Atherly-John 2002^[14]^
urinary tract infection	PO antibiotics	5	Hoberman 1999^[16]^
constipation	suppository/enema	5	Dashshan 1999^[17]^
laceration	sutures	4	Barnett 1998^[18]^
croup	PO steroids	4	Ausejo 2000^[19]^
suspected occult bacteremia	IM/IV antibiotics	3	Bass 1993^[20]^
tonsillitis	PO antibiotics	3	Del Mar 2000^[21]^
urticaria	cetirizine	2	Simons 2000^[22]^
Kawasaki Syndrome	IVIG & ASA	2	Newburger 1986^[23]^
alcohol intoxication	intubation	2	Gausche-Hill 2000^[24]^
generalized tonic-clonic seizure in known epileptic	increase clobazam dose	1	Booth 1998^[25]^
radial head subluxation	reduction by hyperpronation	1	Macias 1998^[26]^
epistaxis	silver nitrate	1	Ruddy 1991^[27]^
behavior problems	referral for counselling	1	Dishion 1995^[28]^
bronchiolitis	racemic epinephrine	1	Menon 1995^[29]^
viral conjunctivitis	polysporin ointment	1	Isenberg 2002^[30]^
laceration	tissue glue	1	Barnett 1998^[18]^
constipation	polyethylene glycol	1	Dashshan 1999^[17]^
dog bite	IV antibiotics & debridement	1	Medeiros 2001^[31]^
burn	delayed graft	1	Desai 1991^[32]^
reflux esophagitis	conservative reflux measures	1	Craig 2004^[33]^
pain (following MVC trauma)	morphine infusion	1	Hendrickson 1990^[34]^
reflux esophagitis	ranitidine	1	Cucchiara 1993^[35]^
varicella zoster	supportive care	1	Klassen 2002^[36]^
atopic dermatitis	hydrocortisone cream	1	Alonso 1999^[37]^
meningitis exposure (H flu)	PO antibiotics	1	Daum 1981^[38]^
dislocated patella	relocation under sedation	1	Nikku 1997^[39]^
**Evidence from Adult Studies**			
migraine headache	metoclopramide	3	Colman 2003^[40]^
proximal humerus fracture	cast & sling	3	Gibson 2003^[41]^
ankle fracture	cast	2	Phillips 1985^[42]^
gastritis	antacids	1	Moayyedi 2003^[43]^
clavicle fracture	sling	1	Andersen 1987^[44]^
prozac overdose (2 hours previous)	supportive care	1	Yeates 2000^[45]^
tibial fracture	internal fixation	1	Wyrsch 1996^[46]^
epididymitis	PO antibiotics	1	Redfern 1984^[47]^
panic attack	sublingual short-acting benzodiazepine	1	Dunner 1986^[48]^
aphthous ulcers	viscous lidocaine	1	Saxen 1997^[49]^
fracture T12 vertebrae	T11–12 instrumentation & fusion	1	Shen 2001^[50]^
aggression	haloperidol & lorazepam	1	Gillies 2001^[51]^
tibial fracture	reduction & cast	1	Abdel-Salem 1991^[52]^
acetaminophen overdose	N-acetylcysteine	1	Brok 2002^[53]^
gastrostomy tube fell out	insertion of foley catheter	1	Kadakia 1994^[54]^
increased intracranial pressure	mannitol	1	Schierhout 2003^[55]^
migraine headache	NSAID	1	Bussone 1999^[56]^

msk = musculoskeletal; PO = by mouth; IV = intravenous; IM = intramuscular, MVC = motor vehicle collision; NSAID = non-steroidal anti-inflammatory; T12 = 12^th ^thoracic vertebrae; IVIG = intravenous immunoglobulin; ASA = aspirin; H flu = Haemophilus influenzae

The most common interventions were analgesics for musculoskeletal pain (17/135 = 12.6%) and inhaled beta-agonist for asthma exacerbations (12/135 = 8.9%); both were supported by pediatric RCTs. Out of the 135 patients who received interventions that were based on level I evidence, 113 patients' interventions (83.7%; CI 76.1 to 89.3) were supported by pediatric RCTs.

The interventions unanimously determined, by the five person committee of pediatric emergency department (PED) physicians, to be worthy of level II evidence status are listed in Table 2. Overall, 7.1% (CI 3.9 to 12.4) of interventions, representing 4.6% of the sample, had face validity worthy of level II evidence.

**Table 2:** Diagnoses and Interventions seen in the PED for which there is Level II Evidence

**Diagnosis**	**Intervention**	**Number of Patients**
Ventriculo-Peritoneal shunt malfunction	Ventriculo-Peritoneal shunt revision	1
cast broken	replace cast	1
multiple phalangeal fractures	volar splint hand and f/u with plastics	1
foreign body in foot	removal of foreign body	2
non-displaced supracondylar fracture	backslab and sling	1
non-displaced olecranon fracture	Cast	1
late lateral condyle fracture	open reduction with internal fixation	1
4th & 5^th ^proximal phalangeal fractures	ulnar gutter	1
abscess lower leg	drainage & antibiotics	1
factor nine deficiency	factor nine infusion	1
Wilm's tumour	radical nephrectomy	1

Including level I and II evidence, 87.5% (CI 81.3 to 91.9) of interventions can be considered evidence-based.

Those interventions which were not considered to be evidence-based (level III evidence) are listed in Table 3. Twelve point five percent (CI 8.1 to 18.7) of interventions (8.0% of the sample) fit into this category.

**Table 3:** Diagnoses and Interventions seen in the PED for which there is Level III Evidence

**Diagnosis**	**Intervention**	**Number of Patients**	**Committee Comments**
femur shaft fracture	closed reduction & spica cast	1	could likely do an RCT of open vs closed reduction
metatarsal fracture	cast	1	one dissentor from the committee
contusion hand	volar slab	1	
sprained digit	aluminum splint	2	would make a good RCT vs. placebo
non-displaced fracture thumb metacarpal bone	thumb splint	1	
cellulitis	PO antibiotics	1	RCT needed for PO vs IV antibiotics
cellulitis	IV antibiotics	3	RCT needed for PO vs IV antibiotics
pneumonia	PO antibiotics	5	need higher quality of evidence as we probably over-treat
pneumonia	IV antibiotics	3	see above comment
intussusception	air contrast enema	1	need an RCT of air contrast vs barium enema
dental abscess	drainage & PO antibiotics	1	
erythema multiforme	reactine (cetirizine)	1	Needs to be studied

## Discussion

The results of this study are important for several reasons. First, we have shown that a substantial proportion of the children seen in the PED do not receive any treatment or intervention. In this sample of 1.4% of all PED visits over one year, 35.9% of children received only reassurance or advice. One of the most common reasons that children were seen in the PED without intervention was upper respiratory tract infection (26%) (data not shown), usually associated with fever. This leaves one wondering whether parental education about benign fever and supportive care for upper respiratory tract infection could help shorten PED wait times. Studies on the effect of education in this area could have very important beneficial effects in terms of costs to the healthcare system.

Our study has revealed that a great majority of interventions in the PED are based on pediatric studies (83.7%). Although this is a positive finding, there are several areas of pediatric medicine, such as migraine treatment and fracture management, for which treatment continues to be based on adult studies. Ideally, we would like to see 100% of pediatric interventions based on pediatric studies.

A comparison of our results to similar studies is shown in Table 4. Interestingly, our total "evidence-based" interventions (level I and II) at 56.1% is substantially less than that found in similar studies in inpatient general medicine [1] (82%) and inpatient pediatrics [5] (75%). However, a closer look reveals that the discrepancy between this and previous studies is not in the number of interventions which involved an RCT (51.5% vs. 53% for Ellis and 31% for Moyer) but in the number of interventions meeting the criteria for level II evidence (4.6% vs. 29% for Ellis and 44% for Moyer).

If one compares what was categorized as level II evidence by Moyer's study with our study, there are important differences. For example, interventions such as IV rehydration and antibiotics for urinary tract infection, categorized as level II evidence in the Moyer study, were classified as level I evidence in our study. This may be a reflection of the four year difference between studies and the accumulation of research evidence during that time. Alternatively, our study involved not only inpatients, but also patients discharged directly from the PED. Therefore, interventions which have been better studied such as antibiotics for otitis media [13] and glucocorticoids for croup [19] were included in our study. There were also differences with respect to those interventions that were considered to be worthy of level II evidence status by Dr Moyer's group and which our group relegated to level III evidence. This may simply be due to the degree of specificity involved in labeling interventions. Antibiotics for pneumonia and cellulitis were two such examples. Although our group refused to agree that intravenous antibiotics were required for these diagnoses, they certainly agreed treating a bacterial infection with some form of antibiotics was completely valid. It is interesting to note that something as simple as inserting the label "IV" or "PO" into the intervention could affect the level of evidence that treatment attained.

**Table 4:** Levels of evidence for interventions across different disciplines

Study Type	Internal Medicine^[1]^	General Practice^[2]^	Inpatient Pediatrics^[5]^	Community Pediatrics^[6]^	Emergency Pediatrics
Level I Evidence (%)	53	31	31	40	52
Level II Evidence (%)	29	50	44	7	5
Level III Evidence (%)	18	19	1	53	8
No Intervention (%)	0	0	24	0	36
Sample Size (number of pts)	109	101	142	247*	262

* although the number of patients included was 247, 629 individual "clinical actions" were studied

The limitations of this study are similar to those found with the studies previously carried out in other disciplines [1-5]. Certainly, there was the potential for the committee to be biased when assigning the level of evidence based on personal knowledge of the cases or personal treatment preferences. This situation was minimized by employing a committee of five physicians and requiring group unanimity for placing an intervention in the level II category. One might argue that all members of the committee had an inclination to prefer to categorize interventions as level II, simply based on their own positions as PED physicians. However, this was not proven true as we had substantially fewer interventions placed in the level II category compared to previous studies.

Another potential limitation is derived from the inclusion of interventions under the category of level I evidence without interpreting the results of the RCT. This was done in order to compare our results to the original study by Ellis which, likewise, did not comment on the content of level I evidence.

Finally, while this was a random sample, it represented only 1% of all PED patients seen over the course of a year, therefore it provides an estimate of the proportion of PED treatments that are evidence-based but may not be representative of the full spectrum of different presenting conditions.

There were many strengths of our study when compared to previous studies. The relative inflexibility of our expert panel in committing interventions to the level II evidence group is one positive feature. Although there must be subjectivity in a process such as this, our team was quite rigorous in applying the criteria for this category and limited what must be assigned to this group accordingly. This is reflected in the lower percentage of interventions ranked as level II evidence by our committee (5% compared to 44% in the Moyer study [5]) than those previous. When consensus surrounding the validity of a treatment without RCT evidence is sufficient to warrant the label "level II evidence", certainly there should be a very limited number of interventions that will fit into this category. Another strength was that our study sample was derived from a full year of PED visits which would account for seasonal variation of presenting diagnoses. This made for a more accurate representation of the most common diagnoses and interventions seen within the pediatric emergency department. We would expect the results of our 1% sample to be relatively generalizable to any tertiary care pediatric centre. Finally, the search for evidence was exhaustive, making it unlikely that relevant RCTs, which had been published at the time of this study, were simply not found during the literature search.

## Conclusion

We have shown that a surprisingly large proportion of patients seen in the pediatric emergency department (35.9%) receive no intervention, while approximately half of patients in this setting receive interventions that are based on evidence from randomized controlled trials (level I evidence). The vast majority of these are derived from randomized control trials containing a majority of pediatric patients. Although our study speaks positively of the breadth of knowledge within the unique environment of the pediatric emergency department, we have also identifed numerous important areas for future study within pediatric research.

## Competing interests

The author(s) declare that they have no competing interests.

## Authors' contributions

TK conceived the study. KW, KC, NW, LH and TK designed the study. KW and KC obtained research funding. KW and KC supervised and conducted the data collection. KW managed the data, including quality control. NW provided statistical advice on study design and analyzed the data. KW drafted the manuscript, and all authors contributed substantially to its revision. All authors read and approved the final manuscript. KW takes responsibility for the paper as a whole.

## Pre-publication history

The pre-publication history for this paper can be accessed here:


